# Structure-activity relationships of quinoidal derivatives against *Trypanosoma brucei*

**DOI:** 10.1016/j.rechem.2026.103701

**Published:** 2026-07-29

**Authors:** Luana A. Machado, Karol R. Francisco, Renata G. Almeida, Yujie Uli Sun, Mai Shingyoji, Alysh M. Mercer, Irving G. Lopez, Rafaela S. Ferreira, Guilherme A.M. Jardim, Conor R. Caffrey, Eufrânio N. da Silva Júnior

**Affiliations:** a Institute of Exact Sciences, Department of Chemistry, Universidade Federal de Minas Gerais, CEP 31270-901, Belo Horizonte, MG, Brazil.; b Center for Discovery and Innovation in Parasitic Diseases, Skaggs School of Pharmacy and Pharmaceutical Sciences, 9255 Pharmacy Lane, MC0657, University of California San Diego, La Jolla, CA 92093, USA; c Department of Biochemistry and Immunology, Institute of Biological Sciences, Universidade Federal de Minas Gerais, 31270-901 Belo Horizonte, MG, Brazil.

**Keywords:** Trypanosoma brucei, Quinoidal, Trypanosomiasis

## Abstract

Quinoidal compounds are a well-established class of redox-active molecules with recognized biological activities, including cytotoxicity and antiparasitic effects. In the context of human African trypanosomiasis (HAT), the demand for novel therapeutic options has encouraged interest in repurposing and further characterizing known compounds. In this study, a set of previously described quinoidal derivatives was evaluated for *in vitro* activity against *Trypanosoma brucei* and cytotoxicity against human embryonic kidney (HEK)293 and hepatoblastoma (HepG2) cells. Several compounds exhibited potent antiparasitic activity; however, with the exception of one compound (**4**), selectivity remained a challenge due to associated cytotoxicity. These findings reinforce the relevance of redox-active scaffolds as privileged structures in the development of trypanocidal agents and highlight the need for further optimization to improve therapeutic windows.

## Introduction

Human and animal African trypanosomiasis (HAT and AAT) are infectious tropical diseases caused by protozoan parasites of the genus *Trypanosoma*. These parasites are transmitted by the bite of the tsetse fly or other blood-feeding insects. HAT, or sleeping sickness, is caused by two subspecies, *T. brucei gambiense* and *T. brucei rhodesiense*. The former causes chronic disease in western and central Africa, and the latter results in acute illness in eastern and southern Africa [1]. The disease is almost invariably fatal unless treated. AAT is caused by different species of *Trypanosoma* (e.g., *T. brucei, T. vivax* and *T. congolense*) and places significant economic constraints on livestock production across 10 million km^2^ of sub-Saharan Africa [2].

Treatment options for HAT are limited and comprise drugs with variable efficacy, a limited spectrum of action (dependent on infecting subspecies and disease stage), toxicity, and the need for parenteral administration and medical supervision during treatment, which can be challenging in rural environments [1a,^3^]. The recently approved oral drug, fexinidazole, offers a simplified treatment regimen for both gambiense and rhodesiense HAT [1a]. Even with this progress, the parasites causing both HAT and AAT have shown the ability to develop (cross) resistance to drugs, which encourages the search for new chemical options [2c,3b].

Phenotypic screening platforms to discover new trypanocides most often utilize *T. brucei brucei*, a non-human-infective model with a sequenced genome and long-standing use in molecular parasitology to investigate key biological targets. This model organism has significantly advanced our understanding of trypanosome biology and facilitated early-stage drug discovery [2c,^4^].

Over the years, our group has developed an extensive research program focused on quinoidal compounds, including naphthoquinone and anthraquinone derivatives, investigating their potential as trypanocidal agents against *Trypanosoma cruzi*. These studies identified several redox-active molecules with promising activity, underscoring the potential of quinoidal scaffolds in the search for new treatments for Chagas disease [5].

Quinoidal compounds stand out as one of the most promising molecular classes with trypanocidal activity [6]. Their structures bear close resemblance to coenzyme Q10, a molecule that has essential roles in protozoan metabolism, particularly within the electron transport chain [7]. Quinoidal substances are believed to disrupt the mitochondrial electron transport chain of parasites, which may account for the broad-spectrum antiprotozoal activity observed for these compounds, given that respiratory inhibition alone can lead to death [8]. Furthermore, quinones can generate free radicals through their interaction with the respiratory chain, most notably reactive oxygen species (ROS).

Here, we reasoned that compounds previously synthesized and tested against *T. cruzi* [5e,^12^,13a] may also display activity against *T. brucei*, providing an opportunity to expand the therapeutic scope of this molecular scaffold. We also screened the same compounds against human embryonic kidney (HEK)293 and hepatoblastoma (HepG2) cells to determine selectivity indices, aiming to identify candidates for further optimization.

## Results and discussion

### Chemistry/synthesis

**Scheme 1.**
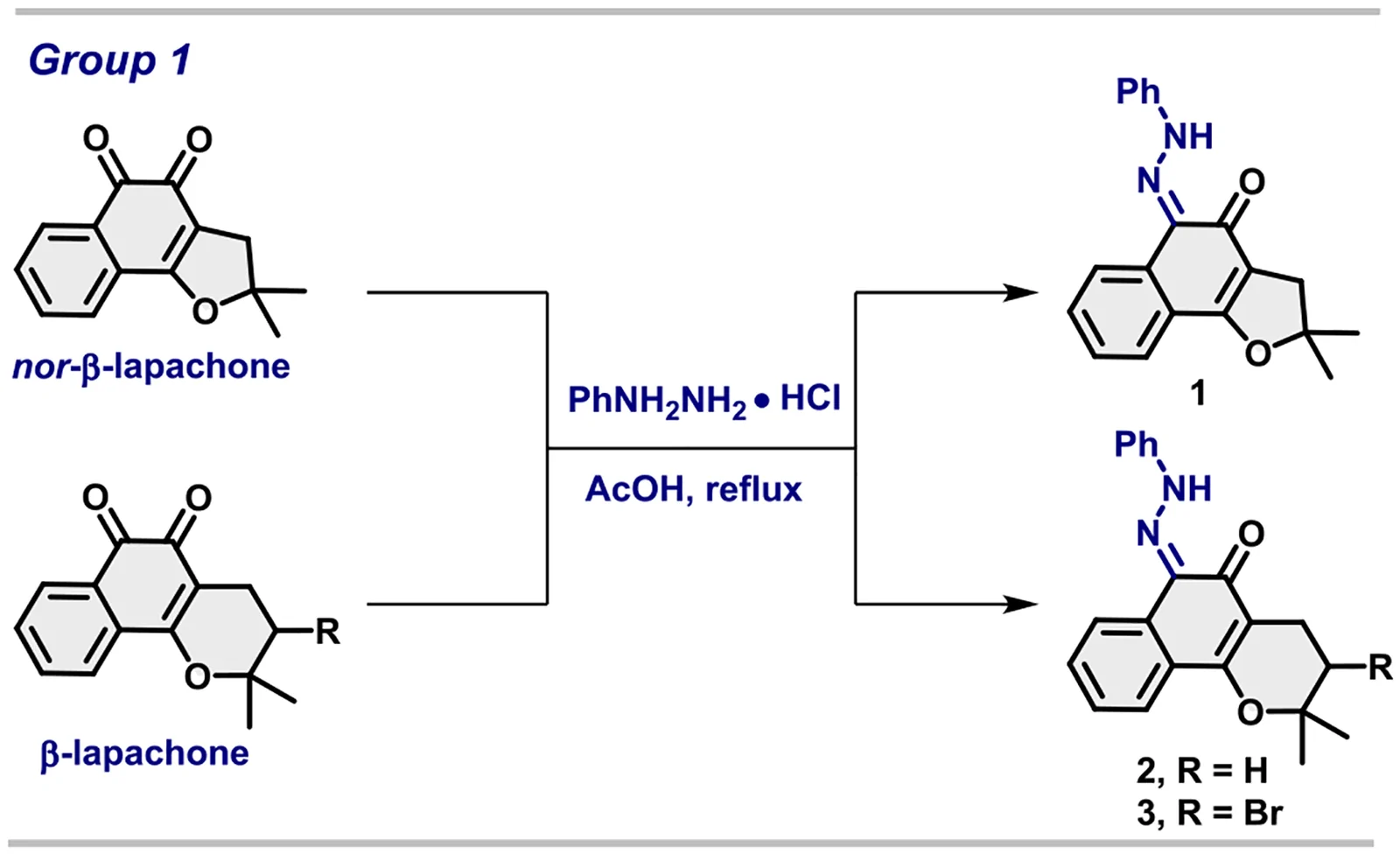
Hydrazone-naphthoquinone hybrids (Group 1).

We evaluated the activity of five quinone groups against *T. brucei*. Lapachones are naturally occurring *ortho*-quinones with notable pharmacological activity [9]. Modification at the redox center of lapachone enables structural optimization for enhanced pharmacological efficacy [10]. In this context, Group 1 comprises hydrazone-naphthoquinone derivatives (**1**–**3**), as shown in Scheme 1, obtained via condensation of *nor*-β-lapachone and β-lapachone derivatives with phenylhydrazine hydrochloride under acidic conditions. This transformation introduces a hydrazone functionality into the quinone framework, resulting in modified naphthoquinone derivatives. Such functionalization may alter the electronic properties of the quinone core and influence its redox behavior, potentially impacting biological activity [11].

**Scheme 2.**
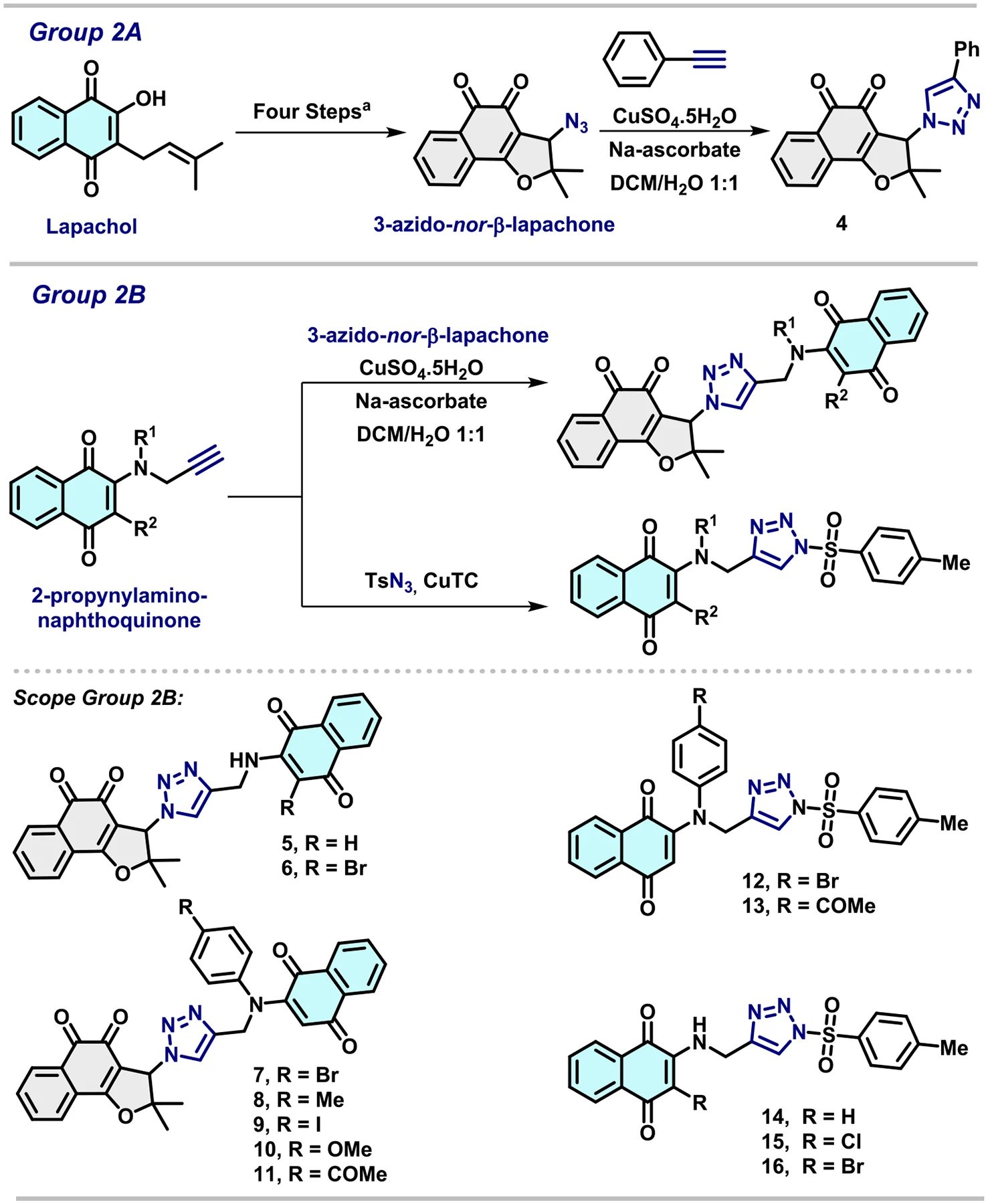
Quinone-triazole hybrids (Group 2). ^a^Ref. [12].

1,2,3-Triazoles are an important class of heterocyclic compounds due to their wide range of pharmacological activities [12]. Thus, the hybridization of a triazole nucleus with quinones can lead to potential drug candidates, combining the bioactive properties of both scaffolds. Group 2 comprises quinone-triazole hybrids and is divided into two subgroups (A and B), as shown in Scheme 2. In Group 2 A (Scheme 2), *nor*-β-lapachone-based 1,2,3-triazole was synthesized starting from naturally occurring lapachol, which was converted into 3-azido-*nor*-β-lapachone via four steps, including oxidation to nor-lapachol, bromine-mediated cyclization to form 3-bromo-*nor*-β-lapachone, and then nucleophilic substitution with sodium azide [12]. A subsequent Cu-catalyzed azide-alkyne cycloaddition (CuAAC) reaction with phenylacetylene afforded the corresponding 1,2,3-triazole derivative (**4**) [12]. This design combines the redox-active quinone core with a triazole moiety, aiming to modulate biological activity through molecular hybridization [13].

Group 2B (Scheme 2) comprises compounds derived from a common 2-propargylamino-1,4-naphthoquinone precursor, which serves as a platform for structural diversification. Following the same CuAAC strategy described for Group 2 A (CuSO_4_/sodium ascorbate), hybrid lapachone-triazole-naphthoquinone derivatives (**5**–**11**) were obtained, further reinforcing the molecular hybridization approach by linking two redox-active quinone units through a triazole spacer [13]. In a complementary approach, quinone-based *N*-sulfonyl-1,2,3-triazoles were obtained via a CuTC-catalyzed reaction of 2-propargylamino-1,4-naphthoquinones with tosyl azide, affording sulfonyl-triazole-tethered aminonaphthoquinones (**12**–**16**). (Scheme 2) [14].

**Scheme 3.**
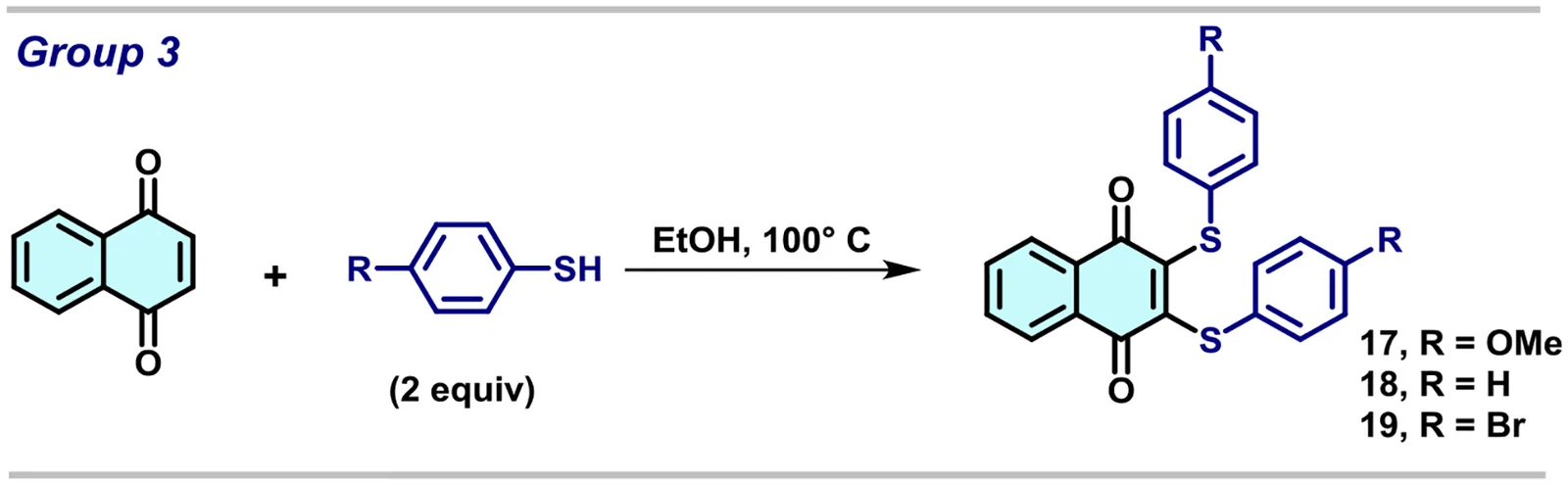
2,3-Bis(phenylthio)-substituted-1,4-naphthoquinones (Group 3).

For Group 3, thionaphthoquinones (**17**–**19**) were synthesized by direct modification of 1,4-naphthoquinone through the insertion of sulfur atoms at the 2- and 3-positions of the quinoidal ring (Scheme 3). The reaction involved nucleophilic substitution at the activated sites of the quinone core using aryl disulfides as sulfur sources, promoting C–S bond formation and affording 2,3-bis(phenylthio)-substituted-1,4-naphthoquinones. The derivatives obtained differ in the electronic nature and substitution pattern of the arylthio groups, enabling fine-tuning of the redox and electronic properties of the quinone scaffold [15].

**Scheme 4.**
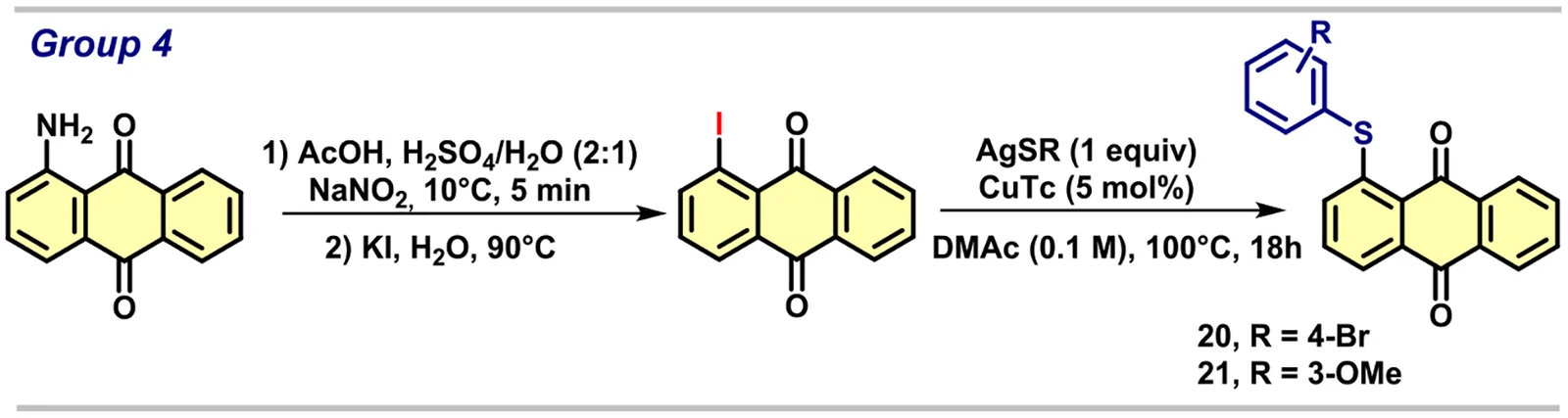
1-thioaryl-substituted-1,4-anthraquinones (Group 4).

In a complementary approach to the sulfur functionalization strategies, Group 4 focuses on anthraquinone derivatives prepared via a distinct synthetic route. Two anthraquinone derivatives were synthesized via a sequential iodination of 1-amino-anthraquinone followed by organoyl-thiolation, facilitating the selective incorporation of sulfur-containing groups onto the aromatic framework. Utilization of silver thiolates (AgSR) in conjunction with a copper source yielded the corresponding sulfur-substituted anthraquinones **20** and **21** (Scheme 4) [16].

**Scheme 5.**
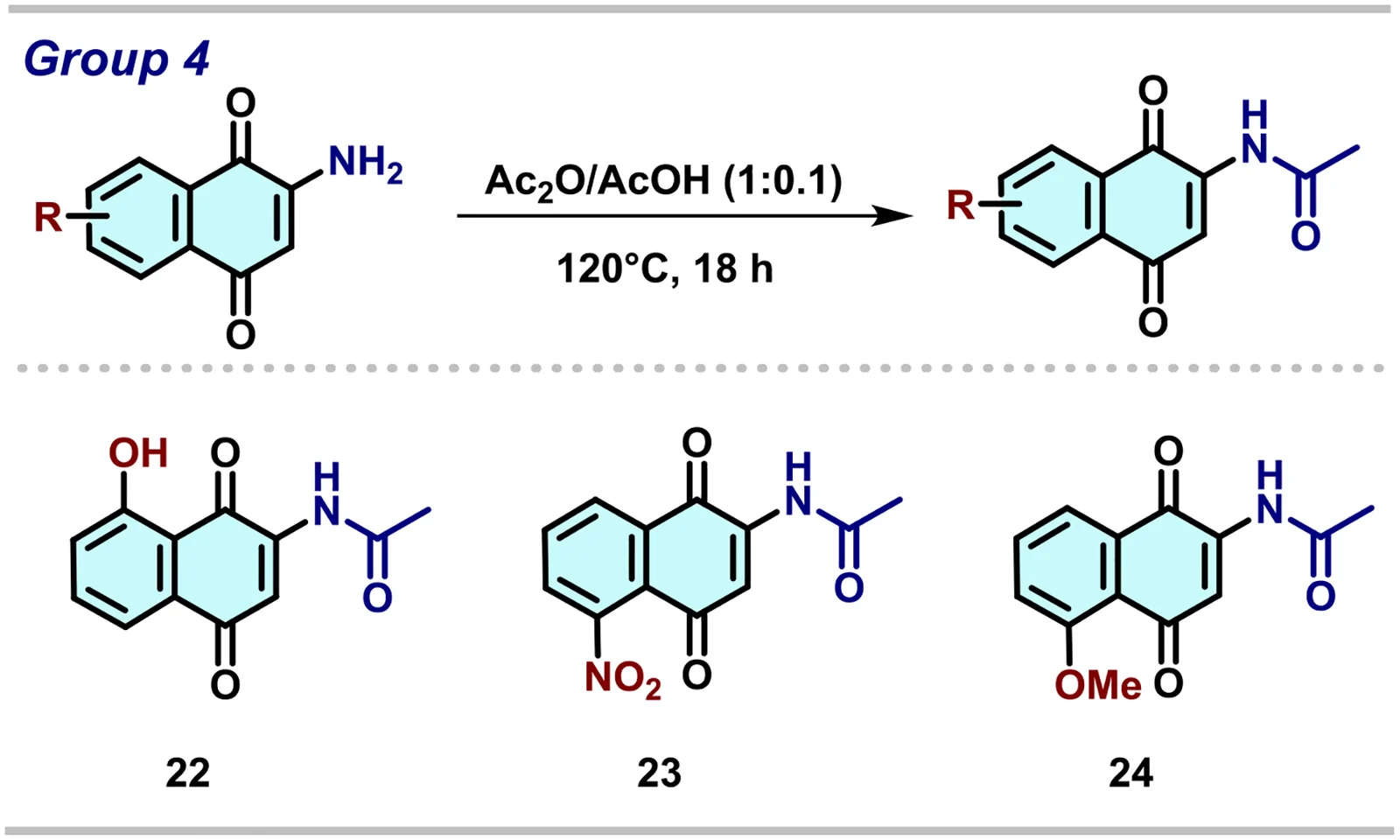
2-acyl-1,4-Naphthoquinones (Group 5).

The last set of compounds, Group 5, was obtained through the acylation of 2-amino-1,4-naphthoquinone derivatives (**22**–**24**), in which the amino group was selectively functionalized with an acyl moiety (Scheme 5). This straightforward transformation afforded a series of acylamido-naphthoquinones, including analogues bearing different substituents at the quinone core (OH, NO_2_ and OMe), to provide structural diversity while preserving the redox-active naphthoquinone scaffold [17].

### Biology

Overall, a panel of 24 compounds was evaluated for their antiparasitic activity against *T. brucei* and their cytotoxicity against HEK293 and HepG2 cells, using a resazurin-based viability assay (Table 1) [18]. Specifically, activity against the parasite and human cell lines was first tested at a single compound concentration, with active compounds then being evaluated in concentration-response assays to measure the concentration at which parasite or cancer cell growth decreased by 50%, i.e., the EC_50_ and CC_50_ values, respectively.

Among the quinoidal compounds evaluated, compounds **1**–**3** (Group 1), **17**–**19** (Group 3), **20** and **21** (Group 4), and **22**–**24** (Group 5) were inactive against *T. brucei*, and, with the exception of compound **22**, were not cytotoxic to HEK293 and HepG2 cells (Table 1). In contrast, compounds from Group 2, which comprise triazole-containing hybrids, displayed distinct biological profiles: compound **4** and derivatives **5**–**11** were active against *T. brucei*, whereas the tosyl-containing derivatives, **12**–**16**, were inactive. Compound **4**, which yielded the lowest EC_50_ value of 0.243 μM, was still 27-fold less potent than pentamidine (0.009 μM), which is a current drug used to treat HAT. Notably, the active compounds incorporate the nor-β-lapachone scaffold, whereas the inactive derivatives are based on a 1,4-naphthoquinone core. The presence of a second quinone moiety introduces an additional redox-active unit but does not enhance antiparasitic potency, while tosyl substitution is detrimental to activity.

Taken together, these observations provide insight into the structural requirements for activity against *T. brucei*. Although all five groups contain quinone-based frameworks, significant antiparasitic activity was observed exclusively for the nor-β-lapachone-derived triazoles (Group 2), indicating that activity is not a general property of redox-active quinones. Moreover, the inactivity of compounds **12**–**16** demonstrates that the presence of a triazole moiety alone is insufficient, highlighting the importance of the nor-β-lapachone scaffold in defining the active motif. Within the active series, incorporation of a second quinone unit (**5**–**11**) did not improve antiparasitic potency relative to compound **4** and was generally associated with increased cytotoxicity. These findings suggest that the nor-β-lapachone–triazole framework represents the key pharmacophoric motif for activity against *T. brucei*, whereas additional quinone functionality primarily increases toxicity.

Among the active compounds from Group 2 (**4**–**11**), all showed significant antitrypanosomal activity at 4 μM, with >90% growth inhibition (Table 1). This prompted further evaluation via concentration-response assays to measure the EC_50_ values, which ranged from 0.24 to 1.00 μM, i.e., potent antiparasitic activity.

The subset of compounds **4**–**11** reveals SAR trends (Table 1). The β-lapachone-triazole hybrid, **4**, that bears the triazole-phenyl ring, was the most potent compound, with an EC_50_ of 0.24 μM. The remaining active compounds each contain two quinone moieties. Comparison of **5** and **6**, which differ only by a single atom on the second quinone (Br vs H, respectively), showed that **6** was slightly more active but also more cytotoxic than **5**. Among compounds **7**–**11**, which differ only in the *para* substituent of an *N*-phenyl group, the following *T. brucei* potency trend was observed: Me > Br > OMe > I > COMe.

Overall, most compounds demonstrated similar cytotoxicity vs HEK293 cells and HepG2 cells, which indicated that these active compounds also exhibited appreciable toxicity, with the respective values ranging from 1.24 to 3.51 μM and 1.13 and 14.0 μM (Table 1). For example, compound **6** exhibited an EC_50_ of 0.47 μM against *T. brucei* but displayed a CC_50_ of 1.33 μM and 1.50 μM against HEK293 and HepG2 cells, respectively, indicating narrow selectivity indices. Similarly, compounds **7**, **8** and **10** demonstrated potent antiparasitic activity but were also cytotoxic, with CC_50_ values between 1.2 and 2.8 μM against HEK293 cells, and 1.1 to 2.6 μM against HepG2 cells (Table 1). Notably, these compounds exhibited comparable cytotoxicity profiles, indicating that differences in selectivity within this subset are primarily driven by variations in antiparasitic potency rather than toxicity. In contrast, compounds such as **4** and **11** were comparatively less cytotoxic, with CC_50_ values of 3.5 μM and 3.3 μM, respectively, against HEK293 cells, and values of 14.0 and 11.1 μM, respectively, against HepG2 cells. Consequently, these compounds, particularly **4**, demonstrated the highest selectivity towards *T. brucei* over human cells. The decreased cytotoxicity observed for the HepG2 cell line may indicate a degree of cell line-dependent toxicity, in addition to parasite selectivity. Nevertheless, the selectivity indices (CC_50_/EC_50_) remained modest across the active series, typically below 10, such that further optimization is warranted.

Most of the other tested compounds did not show significant antiparasitic activity (>70% parasite survival; EC_50_ > 4 μM; Table 1). Some of these compounds, such as **22**, exhibited notable cytotoxicity (CC_50_ values ~1.2–2.2 μM), and no antiparasitic activity, raising concerns about their nonspecific toxicity.

To investigate the novelty of compounds **4**–**11** as antitrypanosomal agents, we incorporated their common substructure, comprising the β-lapachone–triazole core, to search for structurally related compounds in various databases. A search in the Clarivate Cortellis Drug Discovery Intelligence database [19] revealed only 11 compounds with reported biological activity, predominantly associated with anticancer effects, including examples previously reported by our group [13b,^20^] and others [21]. Three of these compounds (**4**–**6)**, have demonstrated antitrypanosomal activity against *T. cruzi* [22], motivating the current evaluation of this scaffold against *T. brucei*. A similar search in the PubChem BioAssay database [23] retrieved 53 compounds that contained the β-lapachone-triazole core and were associated with anticancer activity as well as activity against *T. cruzi*, *Leishmania* spp. and *Mycobacterium tuberculosis*. Notably, no reports of activity against *T. brucei* were found for compounds containing this core in either database, highlighting their novelty in the current context.

Interestingly, compounds **4**–**6** were active against both *T. cruzi* and *T. brucei*. In previous studies against *T. cruzi* trypomastigotes, **4**, **5** and **6** displayed IC_50_ values of 17.3 [12], 80.8 [13a] and 6.8 μM [13a], respectively, whereas in the present study, they exhibited EC_50_ values of 0.24, 0.62 and 0.47 μM against *T. brucei* (Table 1). Although direct quantitative comparisons should be interpreted with caution due to differences in parasite species, developmental stages and assay conditions, these results indicate that the lapachone-triazole scaffold retains activity across distinct trypanosomatids. Moreover, the similar rank order of potency observed between the two species suggests that subtle structural features may differentially influence activity depending on the biological context, providing additional insight into the antitrypanosomal SAR of this chemotype.

Overall, the observed SAR reveals a clear trade-off between potency and cytotoxicity driven by quinone content. Among the active compounds, compound **4** stands out for combining robust antitrypanosomal potency with favorable selectivity indices of 14.4 and 57.6 for HEK293 and HepG2 cells, respectively, a profile attributable to its single quinone moiety and triazole-phenyl substituent. Together with the confirmed chemical novelty of the β-lapachone-triazole scaffold against *T. brucei*, these findings establish compound **4** as the most promising starting point for the development of more selective antitrypanosomal agents.

## Conclusion

A series of 24 quinoidal compounds was synthesized and evaluated against *T. brucei*, identifying quinone-triazole hybrids as the only class with significant antiparasitic activity. These compounds displayed sub-micromolar potency, reinforcing the potential of this scaffold. Compound **4** emerged as the most potent compound with the best selectivity indices (14.4 vs HEK293 and 57.6 vs HepG2). Importantly, its structure provides a suitable platform for further derivatization, enabling the design of new analogues to improve selectivity and biological performance. Furthermore, chemoinformatic analysis revealed the novelty of the β-lapachone-triazole scaffold against *T. brucei*. Overall, these findings establish compound 4 as a promising starting point for the development of new antitrypanosomal agents. Future studies will focus on expanding the SAR of the β-lapachone–triazole series to identify analogs with improved potency and selectivity.

## Methods

### Chemistry

#### General remarks

Unless otherwise stated, all reagents and solvents were obtained from commercial suppliers and used without further purification. The synthesis of the reported compounds was carried out following previously described procedures. Compounds **1–3** [11], **4–11** [12,13], **12–16** [14], **17–19** [15], **20–21** [16], and **22–24** [17] were prepared according to previously published procedures, and their spectroscopic data are consistent with the reported literature values.

### Biological assays

#### T. brucei maintenance and viability assay

*T. b. brucei* bloodstream form parasites (Lister 427) were cultivated in HMI-9 medium [24] at 37 °C in 5% CO_2_ and passaged every 48–72 h to maintain exponential growth. Antitrypanosomal activity was measured using a resazurin viability assay, as previously described [18a,b]. Test compounds were prepared as DMSO stock solutions and dispensed into 96-well, clear polystyrene microplates (1 μL/well, final DMSO concentration 0.5%). Trypanosomes in exponential growth phase were suspended in medium at 2 × 10^5^ parasites/mL. Fresh medium (99 μL/well) was added to the assay plate, followed by the addition of parasites (100 μL/well) to achieve a final density of 2 × 10^4^ parasites/well. Assay plates were incubated at 37 °C and 5% CO_2_. After 72 h, 0.5 mM resazurin (AlfaAesar, Cat. B21187) was added (20 μL/well), and the assay plates were incubated for an additional 2 h at 37 °C. Fluorescence was measured at 531 nm excitation and 595 emission wavelengths using a 2104 EnVision^®^ multilabel plate reader.

Test compounds were initially screened at 4 μM, and those that demonstrated >70% growth inhibition were further evaluated in an eight-point concentration response assay to determine EC_50_ values. All assays were performed in three biological replicates, each in technical duplicate (*n* = 6). EC_50_ values were calculated using a sigmoidal four parameter logistic curve in Prism GraphPad Version 8.0.1. The antitrypanosomal drug, pentamidine, was used as positive control.

#### HEK293 and HepG2 cell maintenance, and cytotoxicity assay

HEK293 and HepG2 cells were cultured in DMEM supplemented with 10% heat-inactivated FBS and 1% penicillin-streptomycin. Cells were kept at 37 °C and 5% CO_2_ and were sub-cultured when 60–90% confluent. Cytotoxicity was determined using a resazurin viability assay as previously described [18c,d]. Compounds were dispensed into 96-well, clear polystyrene microplates (1 μL/well, final DMSO concentration 1%). Mammalian cells were suspended in DMEM at 4 × 10^5^ cells/mL. Fresh DMEM (49 μL/well) was added to the assay plate, followed by the addition of cells (50 μL/well) to achieve a final density of 2 × 10^4^ cells/well. Assay plates were incubated at 37 °C and 5% CO_2_. After 48 h, 0.5 mM resazurin was added (20 μL/well), and the assay plates were incubated for an additional 2 h at 37 °C. Fluorescence was measured at 531 nm excitation and 595 emission wavelengths using a 2104 EnVision^®^ multilabel plate reader.

Test compounds were initially screened at 4 μM, and those that demonstrated >70% cytotoxicity were further evaluated in an eight-point concentration response assay to determine CC_50_ values. All assays were performed in three biological replicates, each in technical duplicate (*n* = 6). CC_50_ values were calculated using a sigmoidal four parameter logistic curve in GraphPad Prism Version 8.0.1. The proteasome inhibitor, bortezomib, was used as a positive control.

#### Chemoinformatic analysis

SMILES for compounds **4**–**11** and for their common substructure, the β-lapachone-triazole core, were obtained by drawing their structures in ChemAxon Marvin Sketch and exporting the corresponding SMILES. The SMILES corresponding to each compound and their common substructure were used as queries for a search for compounds with previously reported biological activity in the Clarivate Cortellis Drug Discovery Intelligence [19] database (https://www.cortellis.com/drugdiscovery/home), against all data available as of December 22, 2025 [23]. The core substructure was also employed as a query against the PubChem BioAssays database (https://pubchem.ncbi.nlm.nih.gov/docs/bioassays) on December 22, 2025, to search for compounds containing this substructure and the related bioassays reported.

## Figures and Tables

**Table 1:** Antitrypanosomal Activity and Cytotoxicity of Tested Compounds.

Compound	% growth inhibition *T. brucei* (4 μM)	*T. brucei* EC_50_ ± SD (μM)	HEK293% cytotoxicity (4 μM)	HEK293 CC_50_ ± SD (μM)	HEK293 SI (CC_50_/EC_50_)	HepG2% cytotoxicity (4 μM)	HepG2 CC_50_ ± SD (μM)	HepG2 SI (CC_50_/EC_50_)
**1**	10.6 ± 9.3	>4	0 ± 3.5	>4	NA^a^	0 ± 5.7	>4	NA
**2**	11.3 ± 12.2	>4	0 ± 3.2	>4	NA	1.3 ± 13.9	>4	NA
**3**	0 ± 19.0	>4	0 ± 1.8	>4	NA	0.5 ± 6.8	>4	NA
**4**	98.4 ± 1.2	0.243 ± 0.043	14.6 ± 2.2	3.505 ± 0.412	14.4	98.4 ± 0.5	13.99 ± 3.56	57.6
**5**	96.9 ± 0.3	0.661 ± 0.083	61.4 ± 6.1	2.832 ± 0.431	4.28	53.8 ± 5.8	2.626 ± 0.247	3.97
**6**	95.3 ± 2.6	0.474 ± 0.082	97.7 ± 0.1	1.325 ± 0.210	2.81	97.8 ± 1.5	1.498 ± 0.325	3.16
**7**	96.9 ± 0.8	0.308 ± 0.076	98.6 ± 0.1	1.238 ± 0.172	4.02	99.4 ± 0.4	2.133 ± 0.054	6.93
**8**	97.3 ± 1.0	0.275 ± 0.047	98.7 ± 0.2	1.335 ± 0.219	4.85	92 ± 2.5	2.371 ± 0.105	8.62
**9**	93.2 ± 3.1	0.553 ± 0.009	99.1 ± 0.2	2.283 ± 0.438	4.13	99.2 ± 0.1	2.400 ± 0.179	4.34
**10**	96.3 ± 1.8	0.385 ± 0.083	99.6 ± 0.4	1.490 ± 0.373	3.87	99.3 ± 0.4	1.127 ± 0.145	2.93
**11**	95.6 ± 1.6	0.996 ± 0.163	97.1 ± 0.8	3.250 ± 0.461	3.26	98.5 ± 0.7	11.10 ± 1.99	11.1
**12**	0 ± 16.3	>4	44.1 ± 0.9	>4	NA	11.5 ± 8.9	>4	NA
**13**	0 ± 9.8	>4	98.5 ± 0.3	>4	NA	19.0 ± 2.9	>4	NA
**14**	0 ± 14.6	>4	0 ± 1.6	>4	NA	0 ± 1.4	>4	NA
**15**	0 ± 13.5	>4	9.2 ± 1.0	>4	NA	0 ± 6.7	>4	NA
**16**	8.7 ± 12.8	>4	16.5 ± 3.0	>4	NA	10.3 ± 9.0	>4	NA
**17**	10.2 ± 4.3	>4	68.9 ± 2.6	>4	NA	0 ± 9.8	>4	NA
**18**	3.1 ± 15.7	>4	45.2 ± 3.8	>4	NA	6.9 ± 11.3	>4	NA
**19**	0 ± 12.1	>4	30.8 ± 1.1	>4	NA	11.9 ± 5.7	>4	NA
**20**	0 ± 7.3	>4	3.9 ± 6.7	>4	NA	3.1 ± 9.5	>4	NA
**21**	0 ± 16.4	>4	6.2 ± 7.3	>4	NA	0 ± 9.4	>4	NA
**22**	0 ± 18.4	>4	94.6 ± 1.1	1.158 ± 0.216	NA	99.4 ± 0.7	2.230 ± 0.415	NA
**23**	0 ± 11.9	>4	98.6 ± 0.1	>4	NA	81.5 ± 11.5	>4	NA
**24**	23.2 ± 20.4	>4	65.4 ± 3.1	>4	NA	4.6 ± 3.6	>4	NA
**Pentamidine**	100.0 ± 3.2	0.009 ± 0.003	NA	NA	NA	NA	NA	NA
**Bortezomib**	NA	NA	100.0 ± 2.6	0.010 ± 0.002	NA	100.0 ± 4.1	0.033 ± 0.002	NA

a NA = not applicable.

## Data Availability

Data will be made available on request.
